# The Role of Genetic Data in Selecting Device-Aided Therapies in Patients With Advanced Parkinson’s Disease: A Mini-Review

**DOI:** 10.3389/fnagi.2022.895430

**Published:** 2022-06-10

**Authors:** Germaine Hiu-Fai Chan

**Affiliations:** Department of Medicine, Queen Elizabeth Hospital, Kowloon, Hong Kong SAR, China

**Keywords:** Parkinson’s disease, genetics, deep brain stimulation, apomorphine, levodopa/carbidopa intestinal gel

## Abstract

Parkinson’s disease (PD) is a common neurodegenerative disease. At present, 5–10% of PD patients are found to have monogenic form of the disease. Each genetic mutation has its own unique clinical features and disease trajectory. It is unclear if the genetic background can affect the outcome of device-aided therapies in these patients. In general, monogenic PD patients have satisfactory motor outcome after receiving invasive therapies. However, their long-term outcome can vary with their genetic mutations. It appears that patients with leucine-rich repeat kinase-2 (LRRK2) and PRKN mutations tended to have good outcome following deep brain stimulation (DBS) surgery. However, those with Glucocerebrosidase (GBA) mutation were found to have poorer cognitive performance, especially after undergoing subthalamic nucleus DBS surgery. In this review, we will provide an overview of the outcomes of device-aided therapies in PD patients with different genetic mutations.

## Introduction

Parkinson’s disease (PD) is the second commonest neurodegenerative disease affecting both motor and non-motor domains (Kalia and Lang, 2015). At present, no treatment is available to stop or slow down disease progression. However, currently available therapies can offer symptomatic relief to the patients (Kalia and Lang, 2015). In general, with the use of oral dopaminergic treatment, their symptoms can be controlled for a few years after symptom onset before developing motor and non-motor complications (Aquino and Fox, 2015; Kalia and Lang, 2015).

Device-aided therapies including deep brain stimulation (DBS), continuous apomorphine subcutaneous infusion (CASI), and levodopa/carbidopa intestinal gel infusion (LCIG) have been used in the management of advanced PD when oral pharmacological treatments are no longer sufficient to control the symptoms or when the patients cannot tolerate the drugs. Nevertheless, not all the PD patients are suitable candidates for these treatments. The inclusion and exclusion criteria of device-aided therapies of advanced parkinsonism are summarized in Table 1 (Defer et al., 1999; Bronstein et al., 2011; Fernandez and Odin, 2011; Volkmann et al., 2013; Worth, 2013; Odin et al., 2015; Trenkwalder et al., 2015; Moro and Lang, 2016; Munhoz et al., 2016; Timpka et al., 2017; Williams et al., 2017; Burack et al., 2018).

**TABLE 1:** Inclusion and exclusion criteria for device-aided therapies of advanced PD.

	DBS (Defer et al., 1999; Bronstein et al., 2011; Volkmann et al., 2013; Worth, 2013; Odin et al., 2015; Moro and Lang, 2016; Munhoz et al., 2016; Timpka et al., 2017; Williams et al., 2017)	CASI (Volkmann et al., 2013; Worth, 2013; Odin et al., 2015; Trenkwalder et al., 2015; Timpka et al., 2017; Williams et al., 2017)	LCIG (Fernandez and Odin, 2011; Volkmann et al., 2013; Worth, 2013; Odin et al., 2015; Timpka et al., 2017; Williams et al., 2017; Burack et al., 2018)
Inclusion criteria	1. Diagnosed of idiopathic PD 2. Good levodopa response 3. Experience troublesome motor fluctuations and dyskinesias 4. Experience significant difficulty with activities of daily living		

Exclusion criteria	**Absolute contraindications**		
	1. A minimal disease duration of 5 years 2. Dementia 3. Uncontrolled psychosis 4. Severe depression with suicidal ideation	1. Failure to tolerate oral dopamine agonists 2. Impulse control disorders/Dopamine dysregulation syndrome 3. Severe dementia 4. Uncontrolled psychosis	1. Relative/Absolute contraindication to percutaneous endoscopic gastrostomy (PEG) tube placement 2. Treatment refractory tremor
	**Relative contraindications**		
	1. Elderly patients (i.e., ≥70–75 years old) 2. Levodopa-unresponsive axial symptoms 3. Conditions that increase surgical risks 4. Poor social support	1. Lack of caregiver to manage the infusion pump	1. Moderate to severe dementia 2. Pre-existing peripheral neuropathies 3. Lack of caregiver to manage the infusion pump

To date, approximately 5 to 10% of PD patients are found to have monogenic form of the disease, with either an autosomal dominant or autosomal recessive pattern of inheritance (Lesage and Brice, 2009). Different types of genetic parkinsonism have their own distinct clinical features (both motor and non-motor symptoms), disease trajectories and treatment outcomes (Hardy et al., 2009; Niemann and Jankovic, 2019). Unlike dystonia (Andrews et al., 2010; Tisch and Kumar, 2021), the current view on the association between genetic mutation and treatment response to DBS surgery remains equivocal in PD. Besides, genetics testing is not routinely included in the pre-procedural workup of device-aided therapies in advanced PD. It is unclear if the genetic background of PD patients will affect their treatment outcomes.

Therefore, the objective of this minireview is to provide an overview of the outcomes of device-aided therapies in patients with genetic parkinsonism and to provide insight on the need to include genetic testing when choosing device-aided therapies for advanced PD patients.

## Results

Twenty-three studies with keywords including “DBS,” “apomorphine,” “levodopa carbidopa intestinal gel,” “PD,” “genetic,” and genes including leucine-rich repeat kinase-2 (LRRK2), SNCA, PRKN, PINK1, and Glucocerebrosidase (GBA) were identified from PubMed search. The results are summarized in Table 2.

**TABLE 2:** Summary of treatment outcomes of device-aided therapies in patients with monogenic parkinsonism.

Author	Study design	Number of subjects	Type of therapy	Results	
**LRRK2 mutation**					
Schüpbach et al., 2007	Case control study	69 • LRRK2 carriers: 9 • G2019S mutation: 8 • T2031S mutation: 1 • Non-mutation carriers (controls): 60	Bilateral STN DBS	M	• Significant improvement in UPDRS II, III, and IV (*off* medications) after surgery • No significant difference between the mutation carriers and non-mutation carriers
				NM	• No change in the cognitive function in both groups after surgery • The patient with T2031S mutation developed hallucination and addiction to levodopa 5 years after surgery
				O	• Both groups achieved a mean of >70% reduction in LEDD
Greenbaum et al., 2013	Case control study	39 • LRRK2 G2019S mutation carriers: 13 • Non-mutation carriers (controls): 26	Bilateral STN DBS	M	• Significant improvement in UPDRS III (*off* medications, *on* stimulation) after surgery • No significant difference between the mutation carriers and non-mutation carriers
				NM	N/A
				O	• Significant reduction in the LEDD after surgery • No significant difference between the two groups
Angeli et al., 2013	Case series	94 • LRRK2 G2019S mutation carriers: 5 • Carriers of other mutations (PRKN and GBA mutations): 25 • Non-mutation carriers: 67	Bilateral STN DBS	M	• Significant improvement in motor function and motor complications after surgery
				NM	N/A
				O	>50% reduction in LEDD following surgery
Sayad et al., 2016	Case control study	27 • LRRK2 G2019S mutation carriers: 15 • Non-mutation carriers (controls): 12	Bilateral STN DBS	M	• Greater improvement in UPDRS III in the mutation carriers when switched from *off* medications *off* stimulation condition to *off* medication *on* stimulation condition • Greater improvement in functional status was found in the mutation carriers when stimulated without medications
				NM	N/A
				O	N/A
Chen et al., 2019	Retrospective case control study	57 • LRRK2 G2385R mutation carriers: 8 • Non-mutation carriers: 49	Bilateral STN DBS	M	• Significant improvement in UPDRS II and III (*off* medication) scores after surgery in both groups • No significant difference between the two groups
				NM	• MMSE remained similar after surgery in both groups
				O	• Significant reduction in LEDD in both groups after surgery
Gómez-Esteban et al., 2008	Case series	45 • LRRK2 R1441G mutation carriers: 4 • Non-mutation carriers: 41	Bilateral STN DBS	M	• Limited improvement in motor function and functional status in the mutation carriers after surgery
				NM	N/A
				O	• Limited improvement in the quality of life in the mutation carriers after surgery
Leaver et al., 2021	Retrospective cohort, case-control study	87 • LRRK2-PD patients with DBS surgery: 13 • LRRK2-PD patients without DBS surgery: 74 *Ad hoc* analysis comparing the effect of DBS surgery between LRRK2-PD and idiopathic PD patients: • LRRK2-DBS group: 9 • iPD-DBS group: 14	STN DBS: • Unilateral: 1 • Bilateral: 8 GPi DBS: • Unilateral: 1 • Bilateral: 3	M	• No significant difference in trajectories following DBS surgery between mutation carriers and non-mutation carriers • Both STN and GPi were found to be effective surgical targets in LRRK2-PD patients
				NM	• Three out of 9 LRRK2-PD patients reported depression after STN DBS surgery but no one reported mood change after GPi DBS surgery
				O	• The LRRK2 mutation carriers were more likely to be referred for DBS surgery because of severe dyskinesia • Significant reduction in LEDD after surgery in both mutation carriers and non-mutation carriers • Greater reduction in LEDD in those underwent STN DBS as compared with those underwent GPi DBS
**SNCA mutation**					
Shimo et al., 2014	Case report	1	Bilateral STN DBS	M	• Marked motor improvement after surgery.
				NM	• MMSE remained stable 2 years after surgery
				O	• 30% reduction in LEDD post-op
Youn et al., 2022	Retrospective observational study	4 • Duplication: 3 • Missense mutation: 1	Bilateral STN DBS	M	SNCA duplication carriers: • Improvement in motor complications (including wearing off and dyskinesia) after surgery Missense mutation carrier: • Despite an improvement in dyskinesia, motor function worsened markedly
				NM	SNCA duplication carriers: • No significant cognitive impairment before and after surgery Missense mutation carrier: Deterioration in cognitive function was noted post-op.
				O	SNCA duplication carriers: •>50% reduction in LEDD after surgery Missense mutation carrier: •<20% reduction in LEDD after surgery
**PRKN mutation**					
Capecci et al., 2004	Case report	1	Bilateral STN DBS	M	• Marked improvement in motor function and motor complications was noted after surgery
				NM	• Better mood post-op • Better control of hallucination with reduction in anti-psychotic dosage
				O	•>60% reduction in LEDD was noted after surgery
Lohmann et al., 2008	Case control study	53 • PRKN mutation carriers: 14 • 2 PRKN mutations: 7 • 1 PRKN mutation: 7 • Non-mutation carriers (controls): 39	Bilateral STN DBS	M	• Motor outcome was similar in the mutation carrier and non-mutation carriers after surgery
				NM	• No significant change in cognitive function after surgery between the mutation carriers and the control group.
				O	• Significantly lower LEDD in the group with two parkin mutations than the non-mutation carriers
Kim et al., 2014	Case control study	9 • PRKN mutation carriers: 3 • Non-mutation carriers (controls): 6	Bilateral STN DBS	M	• No significant difference in the UPDRS II, III and IV following surgeries between the mutation carriers and the controls
				NM	N/A
				O	•>70% reduction in LEDD was achieved in both groups; No significant difference was found in the two groups
Angeli et al., 2013	Case series	94 • PRKN mutation carriers: 9 • 2 parkin mutations: 4 • 1 parkin mutation: 5 • Carriers of other mutations (LRRK2 and GBA mutations): 21 • Non-mutation carriers: 67	Bilateral STN DBS: 2 • Bilateral GPi DBS: 3	M	• Greater improvement in motor score with STN DBS than with GPi DBS • Significant improvement in dyskinesia following surgery with GPi DBS
				NM	N/A
				O	• Reduction in LEDD was found in those who underwent STN DBS • Increment in LEDD was noted in those who underwent GPi DBS
Moro et al., 2008	Prospective observational study	80 • PRKN mutation carriers: 11 • 2 PRKN mutations: 6 • 1 PRKN mutation: 5 • Other mutation carriers (PINK1): 1 • Non-mutation carriers: 68	• Bilateral STN DBS	M	• A mean of 35% improvement in UPDRS III (*off* medication) 3–12 months after surgery • The improvement in motor function was found to be sustained for 3–6 years after surgery. • In long term follow-up (i.e., 3–6 years after surgery), similar degree of clinical improvement was noted in the mutation carriers and the control group
				NM	N/A
				O	N/A
Lim et al., 2021	Case report	1	CASI	M	• Significant improvement in OFF time and dyskinesia • Better mobility with markedly fewer falls
				NM	N/A
				O	• Reduction in oral anti-parkinsonian medication dosage
**PINK1 mutation**					
Moro et al., 2008	Prospective observational study	80 • PINK1 mutation carriers: 1 • Other mutation carriers (PRKN): 11 • Non-mutation carriers: 68	Bilateral STN DBS	M	Short term: •>40% improvement in UPDRS III (*off* medications) Long term: • Sustained improvement in motor function was noted
				NM	N/A
				O	N/A
Balestrino et al., 2021	Case report	1	Bilateral STN DBS	M	Within 1-year post-op: • Marked improvement in motor symptoms and fluctuations 3 years after surgery: • Increase in lower limb dyskinesia/tremor/freezing • Frequent falls • Motor fluctuations still under control
				NM	2–3 years after surgery: • Impulse control disorders: Decrease in compulsive video-gaming; Newly developed hyperphagia • Increased daytime hypersomnolence • Non-motor function remained stable post-op
				O	• 30% reduction in LEDD was found following surgery
Borellini et al., 2017, 2021	Case report	1	Bilateral GPi DBS	M	4 weeks after surgery: • Improvement in motor complications was noted •≥4 years after surgery: • Although motor function remained stable, there was an increase in freezing and painful dystonia
				NM	N/A
				O	6 months after surgery: • LEDD remained stable ≥4 years after surgery: • There was an increment in LEDD
**GBA mutation**					
Angeli et al., 2013	Case series	94 • GBA mutation carriers: 16 • Carriers of other mutations (LRRK2 and PRKN mutations): 14 • Non-mutation carriers: 67	• Bilateral STN DBS: 13 • Bilateral GPi DBS: 2 • Unilateral VIM DBS: 1	M	• Less improvement in motor function and motor complications in those with GPi DBS than in those with STN DBS or VIM DBS
				NM	• More deterioration in cognitive function in those with GBA mutation than in the non-mutation carriers after surgery
				O	• Reduction in LEDD following surgery with all three surgical targets
Weiss et al., 2012	Case series	9 • GBA mutation carriers: 3 • N370S mutation: 1 • L444P mutation: 2 • Non-mutation carriers: 6	Bilateral STN DBS	M	• Sustained improvement in motor function for up to 10 years after STN DBS surgery • Limited treatment response of axial symptoms from STN DBS was noted since 4–6 years post-op
				NM	• All GBA mutation carriers developed cognitive impairment at 2–4 years after surgery
				O	N/A
Lythe et al., 2017	Case control study	34 • GBA mutation carriers: 17 • Non-mutation carriers (controls): 17	Bilateral STN DBS: 15 • Bilateral GPi DBS: 2	M	• No significant difference in the motor function between the GBA mutation carriers and the controls after surgery
				NM	• Cognitive impairment was more common and severe in GBA mutation carriers after surgery • Non-motor symptoms (with cardiovascular symptoms as an exception) were found to be more severe in GBA mutation carriers post-op
				O	• The GBA mutation carriers were found to have a significantly worse quality of life following surgery
Mangone et al., 2020	Retrospective study	208 • GBA mutation carriers: 25 • Non-GBA mutation carriers: 183 • LRRK2 mutation carriers: 22 • PRKN mutation carriers: 18 • Non-mutation carriers: 143	Bilateral STN DBS	M	• Improvement in motor function in the GBA mutation carriers after surgery • No significant difference in motor response between GBA mutation carriers and non-GBA mutation carriers
				NM	• Early cognitive decline was found in the GBA mutation carriers 1 year after STN DBS surgery
				O	• Reduction in LEDD was noted in the GBA mutation carriers 1 year after surgery
Pal et al., 2022	Case control study	366 • GBA+DBS+: 58 • GBA+DBS-: 82 • GBA-DBS+: 98 • GBA-DBS-: 128	Bilateral STN DBS	M	• Improved motor function was noted in GBA mutation carriers after surgery • No significant difference between the GBA mutation carriers and the non-GBA mutation carriers
				NM	• The combined effects of GBA mutation and STN DBS resulted in a negative impact on cognition
				O	• 50% reduction in LEDD in the GBA mutation carriers post-op

*M, motor features; NM, non-motor features; O, other features; UDPRS, Unified Parkinson’s Disease Rating Scale; STN, subthalamic nucleus; GPi, globus pallidus internus; VIM, ventral intermediate nucleus of the thalamus; DBS, deep brain stimulation; LEDD, levodopa equivalent daily dose; and CASI, continuous apomorphine subcutaneous infusion.*

### Monogenic Parkinsonism

#### Autosomal Dominant Monogenic Parkinsonism

##### Leucine-Rich Repeat Kinase-2 Mutation

Mutations in the LRRK2 gene are the commonest mutations in sporadic and familial PD. Patients with LRRK2 parkinsonism share clinical features and disease courses which resemble closely to those with idiopathic PD. They often manifest as late-onset PD with typical clinical features of asymmetrical, tremor-dominant parkinsonism with bradykinesia and rigidity. Dystonia, especially painful off-period foot dystonia, is more common in LRRK2 mutation carriers after starting dopaminergic treatment. They tend to have a lower rate of cognitive impairment and hyposmia (Healy et al., 2008; Tolosa et al., 2020). Sleep complaints are frequently seen. Unlike the idiopathic PD patients, their sleep dysfunction is characterized by frequent sleep onset insomnia with less prominent REM sleep behavioral disorders (Pont-Sunyer et al., 2015). Most of these patients have excellent response to dopaminergic treatment (Healy et al., 2008). Even though they have a similar rate of dyskinesia as the non-carriers, it takes a longer period for them to develop dyskinesia after starting levodopa (Healy et al., 2008).

Among the PD patients with LRRK2 mutations, they were more likely to be referred for surgery because of severe dyskinesia (Leaver et al., 2021). They often responded well to DBS surgery. Schüpbach et al. (2007) showed that subthalamic nucleus (STN) DBS is beneficial in LRRK2-PD patients. Artusi et al. (2019) described that motor function improved by 46% in LRRK2 mutation carriers after surgery. Nonetheless, the treatment outcome can vary with the gene mutation in LRRK2-PD patients. Carriers of LRRK2 G2019S gene mutation, the commonest gene mutation in LRRK2-PD patients, were found to have excellent response to STN DBS and their treatment outcome was not inferior to that of the non-carriers (Greenbaum et al., 2013; Sayad et al., 2016). Besides, sustained benefit was seen in two patients with LRRK2 G2019S mutation 9 years after implantation of STN neurostimulator (Schüpbach et al., 2007). Good surgical outcome was also reported in carriers of LRRK2-G2385R gene mutations, which is prevalent in the Hans Chinese population (Chen et al., 2019). On the other hand, PD patients with R1441G mutation seemed to have unsatisfactory outcome from DBS surgery (Gómez-Esteban et al., 2008).

The majority of LRRK2 carriers had a stable cognitive performance after surgery (Schüpbach et al., 2007; Artusi et al., 2019). However, a patient with T2031S mutation developed hallucination and behavioral disorder with addiction to levodopa 5 years post-operatively (Artusi et al., 2019).

Although most of the studies were conducted in patients undergoing STN DBS surgery Leaver et al. (2021), revealed that both STN and globus pallidus internus (GPi) were effective surgical targets in controlling motor symptoms. However, there were three patients developing depression following STN DBS surgery while none of the patients with GPi DBS implanted experienced mood dysfunction (Leaver et al., 2021).

Reduction in dopaminergic medication dosage was reported in the carriers of LRRK2 mutation, especially in those who underwent STN DBS surgery (Artusi et al., 2019; Leaver et al., 2021).

Furthermore, as LRRK2 mutation carriers are known to be associated with sleep dysfunction (Pont-Sunyer et al., 2015), they may not be good candidates for CASI. As for LCIG therapy, they appeared to be good candidates for this treatment because of good response to levodopa (Healy et al., 2008). Nonetheless, no studies are available for the use of infusion therapy in this group of patients.

##### SNCA Mutation

SNCA mutation was the first gene discovered to cause autosomal-dominant PD. The disease often starts in the fourth or fifth decades. However, one-third of SNCA-PD patients has an early age at onset (i.e., 20–40 years). Atypical features, such as anterocollis/retrocollis, pyramidal signs and alien limb syndrome, can be seen in the SNCA mutation carriers. Moreover, cognitive decline is found in 70% of patients. They often respond well to dopaminergic treatments (Trinh et al., 2018).

The overall treatment outcome of DBS surgery in SNCA-PD patients is satisfactory but it may differ with the type of gene mutation. Shimo et al. (2014) reported that a PD patient with SNCA duplication benefited from STN DBS surgery. Following the surgery, the motor fluctuation improved without developing hallucinations or dementia. Youn et al. (2022) showed that motor fluctuation improved in three PD patients with SNCA duplication after STN DBS surgery. Sustained benefit was observed for up to 10 years post-operatively. There was a significant reduction in the mean levodopa equivalent daily dose (LEDD). Non-motor function remained stable. However, in the patient with SNCA missense mutation (c.158C.A [p.A53E]), although there was motor benefit from the surgery, the patient suffered from natural progression of the disease. She became wheelchair bound and demented 3.5 years after implantation of neurostimulator (Youn et al., 2022).

So far, no case reports are available for the use of CASI and LCIG treatments in SNCA mutation carriers. Infusion pump therapies may be considered in these patients who respond to dopaminergic treatment well and do not suffer from severe dementia.

#### Autosomal Recessive Monogenic Parkinsonism

##### PRKN (Parkin) Mutation

PRKN mutation is frequently seen in patients with early onset autosomal recessive PD and juvenile PD. The disease usually starts in the third and fourth decades. Typical clinical manifestations at disease onset include limb bradykinesia and tremor, as well as symmetrical foot dystonia (Lücking et al., 2000). Besides, these patients tend to develop depressed mood without executive dysfunction or cognitive impairment (Song et al., 2020). In general, they have a good response to levodopa and a slower disease progression. Dyskinesia is common at a later stage of disease (Lücking et al., 2000).

In contrast, there were case reports of atypical presentations of PRKN mutation carriers. Klein et al. (2000) described an Italian family with PRKN deletions presenting as adult-onset tremor-dominant parkinsonism. Gao et al. (2020) reported a Han Chinese family with PRKN mutation suffering from symmetrical resting tremor of lower limbs without rigidity.

In general, the outcome of STN DBS in this group of patients was favorable. Studies reported that motor function improved by 46 to 84% after surgery (Capecci et al., 2004; Lohmann et al., 2008; Kim et al., 2014; Artusi et al., 2019; de Oliveira et al., 2019). Disabling dyskinesia was found to improve or disappear with STN neurostimulation (Capecci et al., 2004; Kim et al., 2014; Artusi et al., 2019). Sustained improvements in motor function was reported 2 to 5 years post-operatively (Lücking et al., 2000). Nonetheless, postural stability and gait was worse in PRKN carriers than in non-carriers after receiving STN DBS (Kim et al., 2014).

Besides, significant decline in cognitive function was not seen in these patients who received STN DBS surgery (Artusi et al., 2019). Medication reduction was reported (Capecci et al., 2004; Lohmann et al., 2008; Kim et al., 2014; Artusi et al., 2019; de Oliveira et al., 2019). Artusi et al. (2019) revealed that the LEDD was reduced by 61% in PRKN mutation carriers after surgery.

Limited data is available for PRKN mutation carriers undergoing pallidal stimulation. A United Kingdom group reported limited motor benefit in this group of patients (21% improvement with GPi DBS vs. 31% improvement with STN DBS). However, motor complications, in terms of UPDRS-IV scores, improved by 70%. In addition, there was in an increment in the dopaminergic medication dosage after undergoing GPi DBS surgery (Angeli et al., 2013).

Furthermore, the type of gene mutation in PRKN mutation carriers may have an impact on the response to DBS surgery. A French group indicated that as compared with the non-carriers and single heterozygous mutation carriers, there was a significant reduction in dopaminergic medication dosage in patients with homozygous or compound heterozygous mutations after surgery (Lohmann et al., 2008). Yet, little information is available concerning the association between surgical outcome and genetic background of PRKN-PD patients and it is premature to conclude if those with 2 PRKN mutations tend to have better treatment response.

On the other hand, CASI therapy may be beneficial in the PRKN mutation carriers, especially when severe cognitive impairment is known to be rare in this group of patients. Lim et al. (2021) reported that a 47-year-old Malaysian woman with PD who carried homozygous PRKN mutation (p.Cys441Arg, c.1321T>C, and exon 12) showing excellent response from CASI treatment.

As for LCIG therapy, there is a lack of literature concerning its use in PRKN mutation carriers. However, this group seldom has marked cognitive impairment or peripheral neuropathies and so they may consider this treatment when motor complications occur.

##### PINK1 Mutation

PINK1 mutation is an important genetic cause of early onset PD. Their clinical manifestations are indistinguishable from that of other early onset PD patients. They usually have a good response to levodopa and a slow disease progression. Some patients may develop dementia at a later stage of the disease (Kasten et al., 2018).

Moro et al. (2008) showed that bilateral STN DBS was beneficial in a patient with PINK1 mutation. Balestrino et al. (2021) reported that a patient with homozygous PINK1 mutation [619C>T-p. (Arg207*)] developed motor fluctuation and subsequently received STN DBS surgery 13 years after disease onset. Transient improvement was noted after surgery. However, 1 year after the surgery, her condition worsened with freezing of gait and dyskinesia (Balestrino et al., 2021). Borellini et al. (2017, 2021) described successful short-term outcome of bilateral pallidal stimulation in a Filipino woman with homozygous L347P PINK1 mutation who suffered from painful dystonia of lower limbs and progressive gait dysfunction. Nevertheless, despite optimal medical treatment and programming, her condition deteriorated due to worsening of dystonia, and she had to walk with aids 4 years after surgery (Borellini et al., 2017, 2021).

So far, no data is available for CASI and LCIG treatments in this group of mutation carriers.

#### Other Genes That Modify Parkinson’s Disease Risks or Outcomes

##### Glucocerebrosidase Mutation

Glucocerebrosidase mutations are the commonest genetic risk factor for the development of PD. A multicenter study conducted in 16 centers worldwide demonstrated a strong association between GBA mutation and PD, especially in the Ashkenazi Jewish population (Sidransky et al., 2009). The clinical features of GBA-PD patients are similar to that of idiopathic PD patients (Winder-Rhodes et al., 2013). Nonetheless, GBA mutation carriers usually present at a younger age (Wang et al., 2014). They tend to have more symmetrical parkinsonian features (Sidransky et al., 2009). They have a more rapid motor progression with a greater impact on postural stability and gait (Brockmann et al., 2015; Davis et al., 2016). Levodopa induced dyskinesia are frequently seen (Lesage et al., 2011). In addition, non-motor symptoms are more common and severe in GBA-PD patients. They include dementia, visual hallucination, REM sleep behavioral disorders, and autonomic dysfunction (Brockmann et al., 2011; Jesús et al., 2016). The progression of cognitive decline is faster in GBA mutation carriers (Brockmann et al., 2015; Davis et al., 2016). Moreover, they tend to require a higher dosage of dopaminergic medications (Brockmann et al., 2015).

The disease trajectory can be affected by the type of GBA variants. Jesús et al. (2016) indicated that deleterious GBA variants (including L444P mutation) were associated with a more rapid progression to dementia and visual hallucination. On the contrary, benign GBA variants (including E326K mutation) tended to develop dyskinesia (Jesús et al., 2016).

As a whole, motor outcome was satisfactory in GBA mutation carriers undergoing STN DBS surgery (Artusi et al., 2019). The UPDRS motor score improved by 49% in these patients after surgery (Artusi et al., 2019). However, post-operative cognitive impairment was found to be more common and severe in this group of patients (Lythe et al., 2017; Artusi et al., 2019; Mangone et al., 2020). Pal et al. (2022) revealed that the combined effects of GBA mutation and STN DBS may increase the risk of cognitive decline (Pal et al., 2022). Also, non-motor symptoms (with cardiovascular symptoms as an exception) were found to be more prevalent and severe after surgery (Lythe et al., 2017). Besides, the quality of life worsened significantly in GBA mutation carriers after receiving DBS surgery, with greatest deterioration in domains such as mobility, activities of daily living, cognition, and communication (Lythe et al., 2017). Furthermore, it appeared that the severity of the type of GBA mutation was not linked to a difference in the rate of cognitive decline after operation (Pal et al., 2022).

In contrast, Angeli et al. (2013) reported 2 GBA-PD patients receiving GPi DBS surgery. Their motor benefit was not as robust as that of those with STN DBS implanted (22% improvement with GPi DBS vs. 40% improvement with STN DBS). Yet, the motor complications (especially dyskinesia) markedly improved after surgery. They were able to achieve a greater amount of medication reduction too (Angeli et al., 2013). In addition, this group showed satisfactory outcome in a GBA-PD patient undergoing DBS surgery of unilateral ventral intermediate nucleus of the thalamus (Angeli et al., 2013).

So far, there were no studies concerning the use of infusion therapy in GBA mutation carriers. Anyway, as cognitive impairment is frequently seen in these patients, they may not be good candidates for CASI and LCIG therapies.

## Discussion and Conclusion

This article offered an overview of the outcomes of device-aided therapies in patients with advanced PD with different genetic mutations. At present, the majority of studies were conducted in patients receiving DBS surgery. Data concerning other therapeutic options such as infusion therapies and lesioning surgeries are lacking.

In general, the short-term motor outcome of most patients with genetic parkinsonism is satisfactory. Their motor function and motor fluctuation improved markedly post-op. However, in patients with certain genetic mutations, their condition may deteriorate with time due to cognitive impairment and levodopa-resistant axial features, even when the pre-operative workup indicated that they were suitable candidates for DBS surgery. For instance, in GBA-PD patients, it has been reported that the long-term outcome was limited by the development of dementia after implantation of STN neurostimulators. In the PINK1 mutation carriers, despite transient therapeutic benefit after surgery, patients were found to develop axial symptoms and dyskinesia, which barely responded to medical treatments and titration of neurostimulator settings. To make things more complicated, variants within genetic mutation matter and LRRK2 mutation is a good example. In general, carriers of LRRK2 mutation are known to respond to DBS surgery very well. Yet, some Spanish LRRK2-PD patients with R1441G mutation were found to have suboptimal response from DBS surgery.

At present, genetic testing is not regularly included in the panel of pre-procedural workup of device-aided therapies of advanced PD. Nonetheless, genetic mutation may affect the long-term effects of these invasive therapies. Even though pre-procedural workup results indicate that patients of certain genetic mutations are good candidates of device-aided therapies, the presence of genetic mutations can influence treatment outcome in the long run. Therefore, it may be reasonable to include genetic testing in advanced PD patients when considering invasive therapies.

Nevertheless, currently available data is not sufficient to recommend or refute the use of device-aided therapies in monogenic parkinsonism because there are many genes responsible for genetic parkinsonism (both causative and risk factor gene) and the phenotypes of different mutations vary greatly. Moreover, the number of patients with monogenic PD in each center is very low and so it is difficult to conduct large-scale trials of invasive therapies in these patients.

This review had a few limitations. Most of the available literature comprises retrospective, anecdotal and short-term studies. Secondly, most of the studies focused on the motor outcome following device-aided therapies. The impact of these treatments on non-motor issues, especially cognitive performance, was often neglected. Thirdly, little information is available concerning the use of infusion therapies and lesioning surgery in patients with monogenic PD. It is uncertain if these patients can have a different outcome with invasive therapies other than DBS surgery.

In conclusion, monogenic PD patients usually have satisfactory motor outcome after receiving invasive therapies. However, their genetic background can affect the long-term outcome of device-aided therapies due to development of axial symptoms and cognitive impairment. In general, patients with LRRK2 and PRKN mutations tended to enjoy good surgical outcome following DBS surgery. Yet, carriers of GBA mutation, especially those with STN DBS implanted, were found to have poorer cognitive performance. Therefore, it may be useful to incorporate genetic data in the management of advanced PD patients. Long term multicenter studies are required in the future in order to draw conclusions of predicting outcomes of invasive therapies based on the genetic information.

## Author Contributions

GC was responsible for designing the study and writing the manuscript.

## Conflict of Interest

The author declares that the research was conducted in the absence of any commercial or financial relationships that could be construed as a potential conflict of interest.

## Publisher’s Note

All claims expressed in this article are solely those of the authors and do not necessarily represent those of their affiliated organizations, or those of the publisher, the editors and the reviewers. Any product that may be evaluated in this article, or claim that may be made by its manufacturer, is not guaranteed or endorsed by the publisher.
